# Associations between Acetaminophen Use during Pregnancy and ADHD Symptoms Measured at Ages 7 and 11 Years

**DOI:** 10.1371/journal.pone.0108210

**Published:** 2014-09-24

**Authors:** John M. D. Thompson, Karen E. Waldie, Clare R. Wall, Rinky Murphy, Edwin A. Mitchell, the ABC study group

**Affiliations:** 1 Department of Paediatrics, The University of Auckland, Auckland, New Zealand; 2 School of Psychology, The University of Auckland, Auckland, New Zealand; 3 Discipline of Nutrition, The University of Auckland, Auckland, New Zealand; 4 Department of Medicine, The University of Auckland, Auckland New Zealand; Chiba University Center for Forensic Mental Health, Japan

## Abstract

**Objective:**

Our aim was to replicate and extend the recently found association between acetaminophen use during pregnancy and ADHD symptoms in school-age children.

**Methods:**

Participants were members of the Auckland Birthweight Collaborative Study, a longitudinal study of 871 infants of European descent sampled disproportionately for small for gestational age. Drug use during pregnancy (acetaminophen, aspirin, antacids, and antibiotics) were analysed in relation to behavioural difficulties and ADHD symptoms measured by parent report at age 7 and both parent- and child-report at 11 years of age. The analyses included multiple covariates including birthweight, socioeconomic status and antenatal maternal perceived stress.

**Results:**

Acetaminophen was used by 49.8% of the study mothers during pregnancy. We found significantly higher total difficulty scores (Strengths and Difficulty Questionnaire parent report at age 7 and child report at age 11) if acetaminophen was used during pregnancy, but there were no significant differences associated with any of the other drugs. Children of mothers who used acetaminophen during pregnancy were also at increased risk of ADHD at 7 and 11 years of age (Conners’ Parent Rating Scale-Revised).

**Conclusions:**

These findings strengthen the contention that acetaminophen exposure in pregnancy increases the risk of ADHD-like behaviours. Our study also supports earlier claims that findings are specific to acetaminophen.

## Introduction

*JAMA Pediatrics* recently published a potentially alarming finding: Acetaminophen taken during pregnancy increased the risk of attention-deficit/hyperactivity disorder (ADHD) symptoms in their 7 year old offspring [1]. The use of acetaminophen in pregnancy led to an increased likelihood of offspring taking prescribed ADHD medications (e.g., methylphenidate) and an increased likelihood of hyperkinetic disorder (a severe form of ADHD). ADHD is the most common neurodevelopmental disorder, affecting over 5–10% of the school age population, and is characterised by inattention, hyperactivity and impulsivity [2]. Despite having an early onset, ADHD is most commonly diagnosed and treated between the ages 7 and 12 years [3] and is reported to persist through to adolescence and beyond in about 80% of cases [4].

The finding that even low doses of acetaminophen (indicated by the number of weeks of drug exposure) can affect behaviour 7 years later is alarming because acetaminophen (paracetamol) is the most commonly used antenatal drug [5]. If supported, understanding the biological mechanism underlying the acetaminophen-behaviour link would likely take precedent in tandem with efforts to inform the general public of the newly identified risks of this commonly used over-the-counter drug. The finding is also noteworthy because of the many strengths of the above study. Firstly, the sample consisted of a large cohort of mothers (64 322 individuals enrolled in the Danish National Birth Cohort) and multiple child outcomes were included (Strengths and Difficulties Questionnaire [SDQ], medication use for ADHD, and hospital records for hyperkinetic disorder. Secondly, the longitudinal research was sampled prospectively, meaning that the results were unlikely affected by recall bias. Finally, the analyses controlled for a wide range of factors that might influence both medication-taking in pregnant mothers (e.g., reported fever, inflammatory problems) and ADHD symptoms in offspring (e.g., birthweight, antenatal smoking and alcohol use).

In an Editorial in the same issue of *JAMA Pediatrics,* Cooper et al. [6] underscore the importance of the acetaminophen findings but also highlight some of the study weakness. They rightly stress that the correlational design of the study (among other factors) does not permit us to infer causation. As such, the authors urge caution when interpreting the study results until the findings are replicated and extended. One such recommendation by the authors to extend the study was to examine other drugs taken during pregnancy to determine if the drug-ADHD association was specific to acetaminophen.

Towards this end, the aim of the present study was to determine the association between drugs commonly taken during pregnancy, including acetaminophen, and ADHD in child members of the prospective Auckland Birthweight Collaborative (ABC) longitudinal study. Aspirin, antacids, and antibiotics were the other drugs, besides acetaminophen, that were analysed in relation to SDQ total difficulties (and sub-scales) at 7 and 11 years of age. We also measured ADHD symptoms with more specificity than the SDQ by using the Conners’ Behavioural Rating Scale: Revised (CRS:R) parent report at ages 7 and 11 years. Our analyses included multiple covariates, including those controlled for in the Danish study. We also included other factors previously shown to be related to SDQ in our study, including antenatal maternal perceived stress. Children of mothers who report antenatal stress have been shown to have a range of poorer neurodevelopmental outcomes, including ADHD [7], [8].

## Methods

### Participants

The Auckland Birthweight Collaborative (ABC) Study has been described in detail elsewhere [9]. In brief, the study originally selected children at birth, between October 1995 and November 1997. Children were selected over the entire time period from the Auckland District Health Board, and from October 1995 to August 1997 in the Waitemata District Health Board. All small for gestational age (SGA) infants (< = 10^th^ percentile for gestation and sex) and a random selection of appropriate for gestational age infants (AGA) were selected during the study, such that the number in each group were approximately equal. Infants were excluded from the study if they were not resident and born in the designated study regions, were preterm, were from multiple births or had congenital abnormalities likely to affect subsequent growth or development.

The study consisted of 1714 children at birth of which 871 had mothers who were self-identified as being of European ethnicity. The study has followed the children at 1 year (via a postal questionnaire), and with face to face assessments at 3.5, 7 and 11 years of age. Only European children were followed up after 3.5 due to the poor response rate for other ethnic groups at the previous time point. At all follow-ups mothers of respondents have been found to be more likely than non-respondents to have a tertiary education (p = 0.05), to be married (p<0.0001), to have high socio-economic status (p<0.0001), not to have smoked during their pregnancy (p<0.0001) and to be older (p = 0.0001). Respondents and non-respondents did not differ in other obstetric and social variables, including the gestational age, birthweight and gender of their infant, parity, type of delivery, maternal social support or maternal stress levels.

At birth, demographics and information about maternal health, perceived stress, drug use, and other lifestyle factors during pregnancy was collected by maternal interview and obstetric records. When children were aged approximately 12 months information about feeding practices, home environment, physical development of the child and maternal parenting stress and social support was collected via postal questionnaire. Children aged 3.5 years were assessed on measures of cognitive and physical development. Mothers were interviewed regarding the child’s health, diet and development during the early years of life and demographic information was collected regarding the child’s family environment, including socio-economic status [10]. When children were aged 7 years, information was again collected from maternal interview about children’s physical activity, diet, behaviour and health. Mothers and children were assessed when the child was aged 11 years. Maternal interview collected demographic information, information about physical activity, diet and health of the child and maternal measures of stress and social support. Children completed questionnaires regarding bullying, self esteem, depression, headaches, behaviour and emotional difficulties.

The study received ethical approval at each phase from the Northern regional ethics committee. Signed consent for the study was given by the parents of the children and assent also given by the child.

## Measures

### Drug Use

Data on paracetemol use was obtained by interviewer-administered questionnaires with the mother soon after the birth of her child. Specifically, ascertainment of drug use was obtained by the following question “Did you have any of these medicines or treatments during your pregnancy”. Amongst this list were: a) anti-inflammatories; b) aspirin based pain killers e.g. Aspro, Disprin) other pain killers e.g. Panadol (acetaminophen); d) antacids.

### Strengths and Difficulties Questionnaire

Symptoms of ADHD were measured at ages 7 and 11 years using the parent format of the Strengths and Difficulties Questionnaire (SDQ) and also using the child format at age 11 [11].

Compared with other child behaviour rating scales the SDQ is considered to be a brief measure of child behaviour which inquires about 25 positive and negative emotional and behavioural attributes. Each child is given a score for 5 subscales each consisting of 5 items. The subscales relate to difficulties in conduct, emotion, hyperactivity-inattention, peer group relationships and pro-social behaviour. Each item is scored on a 3 point Likert scale of 0 = ‘*not true’*, 1 = ‘*somewhat true*’ and 2 = ‘*certainly true*’. The total scores for each subscale are calculated by summing scores on items relevant to a particular problem. The SDQ has a test-retest stability of 0.62 after 4 to 6 months and the internal consistencies of the subscales range from 0.62 to 0.75 [12].

The Total Difficulties score is calculated by summing all four deficit focused subscales (Emotional symptoms; Hyperactivity; Conduct problems; Peer problems). Scores range from 0 to 40. A total of 587 children age 7 years and 614 age 11 years had available data on all five SDQ scales.

### Conners’ Behavioural Rating Scale: Revised - Long Format (CRS:R-L)

The CRS:R-L is a measure of child and adolescent behaviour which is available for children age 6 to 17 years [13]. The CRS: R-L parent questionnaire, used here, consists of 80 items and each item is scored on a four point Likert scale. Responses indicate the extent to which each symptom applies to the target child. Symptoms are scored 0 = ‘*not at all true*’ 1 = ‘*just a little true*’, 2 = ‘*pretty much true’* and 3 = ‘*very much true*’. Three of the CRS:R-L subscales relate to the DSM-IV diagnostic criteria for (1) ADHD Inattentive type, (2) ADHD Hyperactive-Impulsive type, and (3) ADHD Combined type. Three subscales referred to as Conners Global Indices (CGI) are also provided (Restless-Impulsive subscale, Emotional Liability, Total subscale reflecting general problematic behaviour). The Conners’ ADHD Index is used to identify a probable diagnosis of ADHD. Total raw scores for each of the CRS: R-L subscales are summed and converted to T-scores, which are standardised by a child’s age and sex [14].

The CRS-R is one of the most popular of the DSM-IV based rating scales used in research and clinical settings. The parent versions of the CRS:R-L was used here to measure child behaviour, particularly symptoms of ADHD. A total of 575 children aged 7 years had scorable data for the CRS:R-L parent version and 617 children aged 11 years had scorable data.

### Statistical analysis

We analysed SDQ and Conners scores continuously in univariable analysis using t-tests and logistic regression and for multivariable analysis using generalised linear models to control for potential confounders to assess the differences between those with each medication use during pregnancy and those without. Distributions of both the SDQ and Conners were skewed so we also undertook analyses using log transformed data to confirm the results found using the untransformed data.

Multivariable models were controlled for variables that we have previously reported to be related to ADHD symptoms in children and variables considered to be potential confounders in the relationship between medication use and ADHD symptoms.

All statistical analyses were carried out for this paper was generated using SAS/STAT software, Version 9.3 of the SAS System for Windows (SAS Institute Inc., Cary, NC, USA). and were tested with a significance level set at p<0.05.

## Results

We investigated the relationships of the use of acetaminophen (49.8%), anti-inflammatories (1.3%), aspirin (5.3%), antacids (17.4%), and antibiotics (23.5%) in relation to the strengths and difficulties questionnaire at 7 years of age (parent) and 11 years of age (parent and child).

Presented in Table 1 are the descriptive statistics for the SDQ Total Difficulties scores at 7 and 11 years of age by medication taken during pregnancy. We found significantly higher total scores on all 3 SDQ formats if acetaminophen was used during pregnancy, but there were no significant differences associated with any of the other drugs. Additionally to provide a direct comparison with the original analyses we analysed the relationship with the categorical cutoff for defining borderline/abnormal scores. Our power is reduced for this analysis though we found stronger odds ratios at the univariable level than the original study SDQ at 7 (OR = 2.1 95% CI = (0.0, 5.0)), but smaller odds ratios with parent SDQ at 11 (OR = 1.2 95% CI = (0.6, 2.5)) and child SDQ at 11 (OR = 1.0 95% CI = (0.6, 1.6)).

**Table 1:** Means (standard deviations) and differences with 95% confidence intervals (CI) of Strengths and Difficulties Questionnaire (SDQ) Total Difficulties scores at 7 and 11 years of age by medication taken during pregnancy.

	Prevalence	Parent SDQ Total at 7			Parent SDQ Total 11			Child SDQ Total at 11		
		Yes µ (s.d)	No µ (s.d)	Difference 95% CI	Yes µ (s.d)	No µ (s.d)	Difference 95% CI	Yes µ (s.d)	No µ (s.d)	Difference 95% CI
Parecetamol	49.8%	8.0 (5.0)	6.7 (4.6)	**1.2 (0.4, 2.0)**	7.6 (5.2)	6.7 (5.0)	**0.8 (0.0, 1.6)**	10.3 (5.0)	9.3 (5.0)	**0.9 (0.1, 1.7)**
Anti-inflamatories	1.3%	6.2 (3.5)	7.5 (4.9)	−1.3 (−3.5, 0.9)	7.5 (6.8)	7.2 (5.1)	0.3 (−2.7, 3.2)	10.7 (5.3)	9.9 (5.0)	0.8 (−2.1, 3.7)
Aspirin	5.3%	7.9 (5.5)	7.4 (4.8)	0.5 (−1.5, 2.4)	7.8 (6.4)	7.2 (5.1)	0.6 (−1.8, 3.0)	10.2 (5.7)	9.9 (5.0)	0.3 (−1.8, 2.4)
Antacids	17.4%	7.3 (5.0)	7.5 (4.8)	−0.1 (−1.0, 0.7)	7.3 (5.4)	7.2 (5.1)	0.1 (−0.8, 1.0)	9.6 (4.8)	10.0 (5.1)	−0.4 (−1.3,0.5)
Antibiotics	23.5%	7.8 (4.8)	7.3 (4.9)	0.5 (−0.4, 1.4)	7.8 (5.3)	7.0 (5.0)	0.8 (−0.1, 1.7)	10.2 (5.0)	9.8 (5.0)	0.4 (−0.5, 1.3)

We further analysed the relationship of acetaminophen on the sub-scales of the SDQ, in univariable and multivariable analyses (controlling for all potential confounders, see Table 2). Notably the acetaminophen use group had higher scores at each age for all difficulty sub-scales and lower scores for the pro-social scales. We found a consistent effect with the total difficulties scale, though the parental scale at 11 did not quite reach statistical significance at the 5% level. Similarly consistent negative effects were seen for the emotional sub-scale with similar effects for the parental SDQ at 7 and 11. Lower scores were also seen for the conduct subscale on the parental SDQ at 7 and the child SDQ at 11.

**Table 2:** Univariable and Multivariable differences of continuous Strengths and Difficulties Questionnaire (SDQ) scores between the offspring of mothers who took acetaminophen in pregnancy and those that did not at 7 and 11 years of age (95% confidence intervals in parentheses).

	Parent SDQ at 7		Parent SDQ at 11		Child SDQ at 11	
	Univariable	Multivariable*	Univariable	Multivariable*	Univariable	Multivariable*
Total difficulties	**1.2 (0.2, 1.4)**	**1.1 (0.2, 2.0)**	**0.8 (0.0, 1.6)**	0.8 (−0.1, 1.8)	**0.9 (0.1, 1.7)**	**1.1 (0.2, 2.0)**
Emotion	**0.4 (0.1, 0.7)**	**0.4 (0.0, 0.7)**	**0.5 (0.2, 0.8)**	**0.5 (0.1, 0.9)**	**0.3 (0.0, 0.6)**	0.2 (−0.2, 0.6)
Conduct	**0.3 (0.0, 0.5)**	**0.2 (0.0, 0.5)**	0.0 (−0.2, 0.3)	0.0 (−0.3, 0.3)	0.1 (−0.2, 0.3)	**0.3 (0.0, 0.6)**
Hyperactivity	0.3 (−0.1, 0.7)	0.4 (−0.1, 0.8)	0.1 (−0.2, 0.5)	0.1 (−0.3, 0.6)	**0.4 (0.0, 0.7)**	**0.4 (0.0, 0.8)**
Peer Esteem	**0.3 (0.0, 0.5)**	0.1 (−0.2, 0.4)	0.1 (−0.1, 0.4)	0.2 (−0.1, 0.5)	0.2 (−0.1, 0.4)	0.2 (−0.1, 0.5)
Prosocial	−0.1 (−0.4, 0.2)	−0.1 (−0.5, 0.2)	−0.1 (−0.4, 0.2)	−0.1 (−0.4, 0.3)	0.0 (−0.2, 0.3)	0.0 (−0.3, 0.3)

*Controlled for SGA status, sex, age mother left school, maternal smoking during pregnancy, paternal smoking during pregnancy, marital status at birth, parity, socio-economic status, maternal pre-pregnancy BMI, maternal stress in the last month of pregnancy, alcohol consumption in the first trimester, living with the child’s biological father at 3.5 and child activity levels at 3.5, high fever during pregnancy, visiting GP for psychological conditions including depression and anxiety, taking medication during pregnancy for psychological conditions.

Additionally the effects of acetaminophen on the Conners scales showed similar results at 7 years of age (see Table 3). This showed consistently higher (poorer) scores for all but the DSM IV inattentive score and DSM IV inattentive symptoms at 7 years of age. Likewise there were higher scores with all scales for the acetaminophen group for the 11 year old data. However, only the DSM IV- hyperactive impulsive scores at 11 showed a statistically significant difference at the univariable level.

**Table 3:** Univariable and Multivariable differences of continuous parental Conners’ scores between the offspring of mothers who took acetaminophen in pregnancy and those that did not at 7 and 11 years of age (95% confidence intervals in parentheses).

	Parent Conners at 7		Parent Conners at 11	
	Univariable	Multivariable*	Univariable	Multivariable*
Conners ADHD index	**1.6 (0.3, 2.9)**	1.5 (−0.1, 3.0)	0.8 (−0.5, 2.1)	0.5 (−1.1, 2.1)
CGI restless-impulsive	**2.1 (0.7, 3.6)**	**2.1 (0.4, 3.7)**	0.3 (−1.1, 1.6)	−0.1 (−1.7, 1.6)
CGI emotional lability	**2.6 (1.1, 4.1)**	**3.0 (1.3, 4.7)**	1.2 (−0.3, 2.6)	0.6 (−1.1, 2.4)
CGI total	**2.5 (1.1, 3.9)**	**2.6 (1.0, 4.2)**	0.6 (−0.7, 1.9)	0.1 (−1.5, 1.8)
DSM IV inattentive	1.1 (−0.2, 2.4)	0.7 (−0.8, 2.2)	0.6 (−0.8, 2.0)	0.2 (−1.5, 1.9)
DSM IV- hyperactive impulsive	**2.0 (0.5, 3.5)**	**2.0 (0.3, 3.6)**	**1.2 (0.0, 2.5)**	1.3 (−0.2, 2.7)
DSM-IV total	**1.6 (0.3, 3.0)**	1.5 (−0.1, 3.0)	1.0 (−0.3, 2.3)	0.8 (−0.7, 2.3)
DSM inattentive symptoms	0.0 (−0.1, 0.2)	0.0 (−0.2, 0.1)	0.0 (−0.1, 0.2)	0.0 (−0.2, 0.2)
DSM hyperactive-impulsive symptoms	**0.2 (0.0, 0.4)**	**0.2 (0.0, 0.4)**	**0.1 (0.0, 0.2)**	0.1 (−0.1, 0.2)

*Controlled for SGA status, sex, age mother left school, maternal smoking during pregnancy, paternal smoking during pregnancy, marital status at birth, parity, socio-economic status, maternal pre-pregnancy BMI, maternal stress in the last month of pregnancy, alcohol consumption in the first trimester, living with the child’s biological father at 3.5 and child activity levels at 3.5, high fever during pregnancy, visiting GP for physchological conditions includingdepression and anxiety, taking medication during pregnancy for psychological conditions.

## Discussion

In this New Zealand birth cohort study with prospective follow up we found that the children of mothers who used acetaminophen during pregnancy were at increased risk of having symptoms of ADHD as defined by screening questionnaires (the SDQ and Connors Rating System) at 7 and 11 years of age. These findings strengthen the contention that acetaminophen exposure in pregnancy increases the risk of ADHD-like behaviours, as published by Liew et al [1]. Our study also supports the earlier report that the findings are specific to acetaminophen, as there were no associations found with other commonly used drugs in pregnancy (aspirin, antacids and antibiotics).

More specifically, there was a significant effect of acetaminophen use on parent scored SDQ total difficulties at age 7 after controlling for potential confounders. Particularly problematic at age 7 were emotional and conduct problems. Child- but not parent-reported total difficulties at age 11 remained significant in multivariable analyses. Child-reported problems with conduct and hyperactivity/inattention were particularly salient. The hyperactivity-inattention subscale consists of the following items: ‘*restless, overactive, cannot stay still for long’, ‘constantly fidgeting or squirming’, ‘easily distracted, concentration wanders’, ‘can stop and think things out before acting’ and ‘sees tasks through to the end, good attention span’*.

There was also a significant effect of acetaminophen use on parent scored CRS:R-L ADHD symptoms at age 7 after controlling for the potential confounders. These were specific to subscales relating to the DSM-IV diagnostic criteria for ADHD Hyperactive-Impulsive type. Other subscales showing a significant multivariable effect included the CGI Restless-Impulsive subscale (indicates symptoms of restlessness, impulsivity and inattentiveness) and the CGI Emotional Liability subscale (identifies individuals who are prone to emotional responses and behaviours than would be expected to be typical, such as outbursts of crying or anger). Interestingly, for the CRS:R-L the association between acetaminophen use during pregnancy and parent-reported ADHD symptoms was not consistent at 11 years. It is not clear why the finding did not remain significant.

It is also not clear why the SDQ child reported ADHD symptoms remained significant at age 11 but not parent-reported problems. Self-reported problem behaviour has been shown to be a more valid indicator of mental and physical health than parent-reported problems [15]–[17]. It is also well known that ADHD has a complex etiology. It could be that other, more positive and enriching, environmental exposures begin to dilute the neurological outcome of acetaminophen over time. Acetaminophen was hypothesized to act as a hormone disrupter and thus alter fetal brain development [1]. Unfortunately we do not yet have data relating to ADHD symptoms measured after puberty. We also did not have information on dosage of acetaminophen use or trimester of use. Early life acetaminophen exposure may be significant determinants ADHD only at higher doses of the pain killer. In addition, other environmental factors not measured may also act through epigenetics to modify disease risk and neurological outcomes. More research is needed to provide a more precise assessment of risk and consequences of acetaminophen use during pregnancy.

Other limitations should be considered while interpreting our results. Firstly, the follow-up rate of 59 to 70% of the original population is clearly a potential source of bias. Nonetheless, this is unlikely to cause any systematic bias because the percentage of SGA and AGA children remained steady at all phases of the study- at 41and 59% respectively. Secondly, because data was only obtained from European mothers and offspring, generalization of the results should be limited to children of European ethnicity. Thirdly, selection bias may be a problem if one or both of the parents also had ADHD. Unfortunately we have no information on the ADHD status of the parents.

A strength of this study is that the longitudinal design of the research was sampled prospectively and the analyses controlled for a wide range of factors that might influence medication-taking in pregnant mothers (e.g., reported fever, inflammatory problems) and ADHD symptoms in offspring (e.g., birthweight, antenatal smoking and alcohol use). Interestingly controlling for all these factors had little effect on the size of the effect. In addition to the Danish study, we were also able to support the outcomes of the SDQ with more specificity by using the CRS-R parent report at both 7 and 11years. We were also were able to control for the effect of antenatal perceived stress.

There is a balance of weighing up the risk associated with taking acetaminophen during pregnancy and that of not taking it in the presence of potentially serious conditions such as maternal fever. In this study the reported prevalence of maternal fever in pregnancy was less than 5%, compared to approximately 50% who took acetaminophen during pregnancy.
